# Agricultural Mitigation Strategies to Reduce the Impact of Romaine Lettuce Contamination

**DOI:** 10.3390/plants13172460

**Published:** 2024-09-03

**Authors:** Walid El Kayal, Linda Darwiche, Yasmine A. Farhat, Mariane Hdeib, Roaa AlJardaly, Mostapha Shbaro, Christelle F. Iskandar

**Affiliations:** 1 Department of Agriculture, Faculty of Agricultural and Food Sciences, American University of Beirut, Riad El-Solh, P.O. Box 11-0236, Beirut 1107-2020, Lebanon; we21@aub.edu.lb (W.E.K.); yasafarhat@gmail.com (Y.A.F.); mh384@aub.edu.lb (M.H.); ra475@aub.edu.lb (R.A.); mms136@mail.aub.edu (M.S.); 2 Department of Nutrition and Food Sciences, Faculty of Agricultural and Food Sciences, American University of Beirut, Riad El Solh, P.O. Box 11-0236, Beirut 1107-2020, Lebanon; lwd05@mail.aub.edu

**Keywords:** bacterial contamination, *E. coli*, *Salmonella*, heavy metal contamination, romaine lettuce, agricultural practices, irrigation systems, environmental contamination

## Abstract

Background: Leafy greens, particularly romaine lettuce, are often associated with outbreaks due to their susceptibility to contamination from various environmental sources. This study aimed to evaluate the presence of *E. coli*, *Salmonella*, copper, nickel, zinc, and manganese in irrigation water, lettuce leaves, and agricultural soil in the Litani River Basin (LRB), Lebanon. Method: Samples were collected from five demonstration plots employing different agricultural practices. Heavy metal concentrations were determined using atomic absorption spectrometry, while *E. coli* and *Salmonella* testing were conducted through conventional culturing techniques. The impact of *E. coli* contamination on seed germination and the interaction effects between *E. coli* and heavy metals were also examined. The study also compared the effectiveness of various irrigation systems in reducing bacterial contamination. Results: The results demonstrated that contamination levels varied significantly across the plots and irrigation types. This variation underscores the necessity of site-specific mitigation strategies to enhance food safety. Our findings highlight the importance of selecting appropriate irrigation methods and implementing tailored agricultural practices to minimize the risk of contamination. Conclusion: This research provides valuable insights for optimizing agricultural practices in the LRB to ensure food safety and environmental sustainability.

## 1. Introduction

Foodborne illnesses are a concern nowadays, causing more than 200 diseases ranging from diarrhea to cancer. Foodborne diseases cause infections or intoxication due to the presence of pathogens or toxic chemical substances. Those contaminants can naturally occur in the environment or be considered pollutants [1]. Although vegetables are an important part of a healthy diet, leafy greens can sometimes be contaminated with biological and chemical substances [2] because they are grown in open fields and exposed to different sources of contamination, such as fertilizers, unfermented manure, contaminated irrigation water, and contact with wild animals that excrete their feces in the cultured field [3]. Contamination of vegetables is very common worldwide [4,5,6,7]. In Lebanon, microbes [8] and heavy metals [9] were detected in leafy greens. Although many initiatives took place and surveillance programs were implemented [10,11], contamination of leafy greens remains a major public health concern. In fact, a recent study in the US showed leafy greens were associated with 9.18% of foodborne illnesses, contributing to an economic cost of up to USD 5.278 billion annually. Notably, romaine lettuce was associated with 60% of leafy outbreaks and 70% of the costs [12]. Among the contaminants, Enterobacteriaceae were among the most common foodborne pathogens found in leafy greens. It was found that those contaminants can enter the plant tissues and remain inside the leaves even after washing and sterilization, causing food poisoning outbreaks [13,14,15].

Enterobacteriaceae are a group of Gram-negative bacteria involved in many cases of food poisoning. Two well-known strains are *Escherichia coli* and *Salmonella* sp. These fecal pathogenic bacteria cause life-threatening infections in immuno-compromised persons [16]. According to the Centers for Disease Control and Prevention (CDC), leafy greens, including lettuce, are common sources of *E. coli* outbreaks. In 2020, 58.1% of the *E. coli* O157:H7 illnesses were attributed to raw leafy vegetables [17]. In Lebanon, 51.5% of the lettuce samples collected from the Bekaa Valley were found to harbor *E. coli* [8]. In addition, *Salmonella* Typhimurium was the reason for an outbreak in 2021 linked to packed leafy greens in the USA. It was reported that the irrigation water was one of the sources of contamination. This contamination caused illness to thirty-one persons and the hospitalization of four [18]. In Lebanon, 6.37% of the raw vegetables were contaminated with *Salmonella*, possibly originating from the irrigation water [19]. It should be highlighted that the sources of the contamination were detected to be the bad agricultural practices adopted by the farmer at the pre- and post-harvest stages. The lack of clear hygien guidelines for processing and retailing, and the washing step were shown to be major sources of pathogen transmission to fresh produce [20].

Furthermore, metal contamination of the environment and food is also of paramount concern due to its impact on human health. They are required to maintain certain functions in small amounts, but in higher amounts, they become toxic [21]. Alzheimer’s disease, intellectual disability in children, DNS (Deviated Nasal Septum), insomnia, and kidney and liver diseases are among the serious health risks that consumers might be exposed to due to the consumption of heavy-metal-contaminated vegetables [22,23]. In Lebanon, leafy greens were found to harbor high levels of Heavy Metals (HM) [9]. Due to the uncontrolled industrial discharges in the river used for irrigation, 17% of the ground and 14% of the surface water in the region are heavily contaminated by chemical pollutants [24]. Metals will accumulate in the soil to become one of the major sources of leafy vegetable contamination [25,26]. HMs that are bioavailable in the soil will be absorbed through the roots and accumulate in the edible leaves [27,28]. Plants have developed various mechanisms for metal uptake and transfer from the soil to root cells through various transporters. The plant can use metal transport detoxification techniques, chelation, and sequestration into the vacuole to control accumulated metals [29].

In our study, we performed microbiological (*E. coli* and *Salmonella*) and chemical (copper (Cu), nickel (Ni), zinc (Zn), and manganese (Mn)) screening of irrigation water, lettuce leaves and agricultural soil around a highly contaminated agricultural area, the Litani River Basin (LRB) in Lebanon [30,31,32]. Bacterial pathogens in raw leafy greens, especially lettuce, are becoming a major public health concern in Lebanon due to antibiotic resistance [33,34]. In addition, despite the laws and surveillance of the legal authorities, the excessive use of chemical fertilizers and pesticides is widespread in Lebanon, causing the chemical contamination of food [35]. It is thus necessary to track the source of the contamination, whether it originates from the agricultural soil or the irrigation water, to address the problem for a better food safety system. An in vitro analysis was performed to understand the HMs’ effect on the soil’s bacterial response as both contaminants were co-present. In addition, the study evaluated the effect of distinct irrigation systems on bacterial contamination. This evaluation is important to guide farmers in optimizing agricultural practices aligned with environmental stewardship. This study sought to deepen our understanding of the complex ecological challenges within the LRB, but it also aimed to offer practical solutions. By addressing the impact of irrigation systems on bacterial contamination and comparing the bacteria levels in the soil, water, and lettuce across the three systems, our research aimed to guide ecologically responsible land and water resource management in this vital region. It could serve as a reference for authorities to manage contamination during cultivation and after harvesting by highlighting the importance of good agricultural practices and adopting a proper irrigation system and clean irrigation water. This project also shed light on the chemical quality of the crops to better manage the use of fertilizers.

## 2. Results and Discussion

### 2.1. Screening for E. coli and Salmonella

The irrigation water, soil, and lettuce microbiological tests did not detect *Salmonella* sp. Although the prevalence of *Salmonella* in raw greens is high in many countries [36,37,38], this result is consistent with different studies in Lebanon, where *Salmonella* was either not detected or found in few samples of leafy greens [20,39]. However, *E. coli* was found in certain samples (Table 1). During the pre-planting season in D1, an open field, only water collected before planting was contaminated by *E. coli*, although no contamination was observed in soil samples. No *E. coli* contamination was observed in the soil, lettuce, or water after harvesting. In D2, where cultivation took place in greenhouses, the water source was contaminated with *E. coli* during both the pre-planting and harvest seasons; however, no *E. coli* contamination was observed in the soil or lettuce during these seasons. Similarly, in D3, water samples were contaminated with *E. coli* during the pre-planting season, while soil samples remained uncontaminated. However, the contamination pattern shifted during the harvest season, as water samples were no longer contaminated, and soil and lettuce samples exhibited *E. coli* contamination. In D4, before planting lettuce, the microbiological analysis of the soil indicated that soil samples from G1 and G3 were not contaminated with *E. coli*, and those collected from G2 and G4 tested positive for *E. coli*, even though the water sample tested negative for *E. coli*. At harvest, the soil and lettuce remained contaminated in G2 and G4 but not in G1 and G3. However, the water sample was *E. coli*-free. In D5, an open field setting, the water sample tested negative for *E. coli* before lettuce planting, while the soil sample tested positive. However, water, lettuce, and soil samples tested positive for *E. coli* at harvest.

The observed *E. coli* contamination in the water, soil, and lettuce across the five demonstration plots suggested a potential link to current agricultural practices. It was obvious that the water samples collected during the fall were all contaminated. This could be explained by the fact that *E. coli* concentrations in the summer increase to a peak in autumn [40]. This temporal trend may be caused by numerous reasons, such as (1) an increase in temperature, which leads to the evaporation of water and, thus, the concentration of *E. coli* [41,42], (2) *E. coli*’s preference for warm temperatures [43], (3) the increase in the domestic use of water in the summer and thus more sewage disposal [44], (4) the dilution of river water in the winter due to rainfall [45,46], and (5) extremely low temperatures that might stress the bacteria, causing them to likely become Viable But Non-Culturable (VBNC) [47]. Different types of stresses, such as extreme temperatures and toxic HMs, can trigger the VBNC state [48]. Therefore, the extreme temperatures of the LRB (i.e., low during the winter and high during the summer) and possible HM contamination may explain the absence of *E. coli* in the soil and water when potential sources of contamination were detected.

Furthermore, the region’s fertilizers and regulations are poorly enforced, leading to frequent contamination. Farmers frequently use excessive amounts of non-fermented manure, which causes concerns about contaminating the soil with pathogenic bacteria that might reach the lettuce, thus exposing consumers to potential health risks and threatening food safety [49]. Microbial contamination in manure poses a significant threat to public health and agriculture in Lebanon. Studies have highlighted that manure, especially when inadequately managed, can harbor dangerous pathogens like *E. coli* that are found to be, in some cases, antibiotic-resistant [50]. They can infiltrate water sources and agricultural produce. For instance, research on the Litani River, Lebanon’s most polluted river, revealed a high prevalence of fecal contamination and antibiotic-resistant bacteria, underscoring the risks associated with the improper use of manure in agricultural practices. Additionally, the misuse of manure in Lebanese farming systems has been linked to the contamination of leafy vegetables, which can transmit infections to consumers [51]. These findings highlight the urgent need for improved manure management and stricter regulations to protect Lebanon’s public health and the environment. The scenario was observed in the soil collected from D4 and D5, where farmers used unfermented manure prior to the planting season. Therefore, soil and lettuce samples tested positive. 

Romaine lettuce can be exposed to animal feces and contaminated water [52]. Consuming *E. coli*-contaminated lettuce imposes a potential food safety risk as this type of produce is consumed raw without any boiling or cooking to kill the bacteria, and washing in water alone is not enough to remove the bacteria [53]. In our study, lettuce in three of five demonstration plots was contaminated with *E. coli.* In plots where soil samples tested positive for *E. coli* during the harvest season, lettuce samples also tested positive, meaning that *E. coli* was likely transmitted from the soil to the lettuce.

### 2.2. Quantification of Heavy Metals

Historically, the biological contamination of the LRB has been accompanied by chemical contamination of HMs because of human interference originating from municipal wastewater and agriculture discharged into the river [54]. They pose significant health risks and environmental integrity risks. Understanding the levels and distribution of HM contamination in various environmental matrices is crucial for assessing potential health and ecological impacts. In soils, we specifically focus on the bioavailable fraction of HMs as, for many metals, the fraction of bioavailable HMs is relatively small compared with the total concentrations of those metals incorporated into the soils. Each HM exhibited temporal and spatial variability (Table 2).

Soil-bioavailable Ni remained spatially consistent over time, with D1–3 maintaining significantly higher concentrations of DTPA-Ni than fields D4–5. However, this trend is nearly inverted in plants, where lettuce collected from D5 had significantly greater concentrations of Ni than lettuce collected from all other fields besides D4. Lettuce from D4 had significantly greater concentrations of Ni than lettuce from D2, although it was statistically indistinguishable from D1 and D3. These findings indicate a clear disconnect between soil availability and plant uptake. Differences in Ni speciation may partially explain this. For example, soluble Ni is primarily taken up through passive cation transport, which is highly influenced by soil-solution concentrations. Alternatively, if most soil Ni is instead chelated to other ligand compounds, then plant uptake can be more tightly regulated and less determined by pure mass flow [55]. Additional explanations include Ni immobilization in the soil [56] or the possibility that the bacteria in the rhizosphere prevented its movement [57].

Bioavailable concentrations of Zn in the soil were less temporally consistent than Ni. Originally, at pre-planting, most soils exhibited similar levels of bioavailable Zn, except D4, which had significantly lower concentrations of DTPA-Zn compared to other sites (0.80 ppm). The concentration of DTPA-Zn observed in D4 is below the recommended limits for DTPA-Zn and meets the criteria for Zn deficiency [58]. At the final time point, soils from both locations in Talya (i.e., D2 and D3) nearly doubled their concentrations of bioavailable Zn compared to the pre-planting time point. They had significantly greater concentrations of DTPA-Zn relative to all other fields. Common causes of this large increase could include fertilization of either Zn itself or organic matter [58] or changes in the soil pH, which might result from several field-management practices [59]. Concentrations of DTPA-Zn in soils from fields other than D2 and D3 were relatively consistent with concentrations at pre-planting; D4 still maintained the lowest concentration of bioavailable Zn (0.78 ppm).

Counter-intuitively, concentrations of lettuce collected from D4 were also greater than lettuce collected from D1, D3, and D5 despite having significantly lesser bioavailable Zn throughout the growing season, as amounts were low enough to be considered Zn-deficient. However, the highest observed concentrations of Zn by a wide margin were from lettuce collected from D2 (mean = 170 ppm). While no specific Maximum Contaminant Level (MCL) exists for Zn in food, the FAO/WHO recommends a dietary limit of a Provisional Maximum Tolerable Daily Intake (PMTDI) of 0.3–1 mg/kg body weight (BW). Assuming an adult body weight of 60 kg, a regular salad with 106 g of lettuce from D2 from this field could surpass the PMTDI limit. Moderate contamination of Zn in vegetables was reported in levels averaging 86.5 ± 60 mg/kg, thus falling within our observed range [60].

Soil concentrations of bioavailable Cu were relatively low at all sites and much lower than concentrations of other HMs; field averages range between 0.94 and 1.93 ppm. D1 had the lowest average concentrations of Cu at both the pre-planting and final time points (mean = 0.94 and 1.05 ppm, respectively). DTPA-Cu concentrations from D1 were only consistently statistically lower than those of soils from D5, and they were not significantly different from soils from D4 at either time point. Beyond the currently accepted PMTDI by the FAO/WHO (0.05–0.5 mg Cu/kg-BW), the only relevant MCL for Cu in vegetables is that established for Cu concentrations in fats and oils, which range between 0.4 and 0.1 ppm depending on the purity of the oil [61]. While the levels of Cu observed in lettuce within this study (average = 37.7 ± 20 ppm) were consistent with other studies from other MENA regions [62,63,64], they surpassed measurements of Lebanese vegetables conducted previously [60]. Additional regulatory guidance is needed to understand the upper-threshold limits for safe Cu concentrations in vegetables more broadly and leafy greens specifically given their propensity to accumulate higher concentrations of HMs [65].

There was very little observed change in the concentrations of bioavailable Mn between the pre-planting and final time points. The highest levels of soil-bioavailable Mn were significantly observed in D2 and D3, while the lowest levels were found in D4. After harvest, Mn levels remained significantly higher in D2 and D3 than in D4 (which retained the lowest levels). D5 also exhibited low Mn levels, similar to the pre-planting season. The concentrations of Mn accumulated in lettuce were generally much greater than the bioavailable concentrations observed in the soil. Despite having consistently low concentrations of DTPA-Mn, lettuce from D4 had significantly greater concentrations of Mn than lettuce collected from all other sites (163 ppm). Lettuce from D1 had significantly lower concentrations of Mn than lettuce collected from all other sites (61.7 ppm).

When taken together, some overlapping spatial patterns emerge. Soils from D2 and D3 in Talia, the furthest upstream sites within the LRB, tended to be among the more contaminated soils for bioavailable heavy metals (e.g., Ni, Zn, Mn). Soil from D4, the furthest downstream of the sites measured in this study, tended to be less contaminated with these same metals. These observations indicate that natural processes, such as weathering or sediment deposition, likely govern the concentrations of these HMs in soils. These spatial patterns in the soil did not translate to leaf tissue, where no correction was observed between soil and plant tissue concentrations. Lettuce from D3 was never among the most contaminated in any of the elements measured, and lettuce from D4 was never among the least contaminated. The disconnect between soil and tissue concentrations points to the idea that the accumulation of these trace elements is primarily a result of each element’s specific plant uptake pathway rather than soil bioavailability. In fact, from the roots to the leaves, HM molecules should be transported by specific transporters using different systems, such as the active or passive pathways. Once in the cytosol, the plants have a detoxification system that excretes the molecules to keep the concentration below the toxicity level [66].

Furthermore, it is known that there is a relationship between soil pH, Soil Organic Matter (SOM), and plants’ absorption of metals. In Lebanese soils, which typically have a pH ranging from 7 to 8 [67], the slightly alkaline conditions generally reduce the bioavailability of micronutrients like manganese (Mn), copper (Cu), and zinc (Zn) by decreasing their solubility [68]. SOM can mitigate this by forming chelates that enhance metal availability despite pH limitations [69]. This relationship was evident in our demo plots, where the highest Zn bioaccumulation in the leaves (BAC = 57.95) in demo 4 aligned with the highest pH (7.6) and relatively low SOM (2.7%), suggesting a complex interaction between pH and SOM. Similarly, lower Ni (BAC = 0.47 in D2 and BAC = 1.13 in D3) and Mn (BAC = 1.18 in D2 and BAC = 0.93 in D3) levels were observed with lower pH (7 in D2 and 7.1 in D3) and higher SOM (3.21 in D2 and 2.18 in D3) (Supplementary Table S1).

### 2.3. Influence of E. coli Contamination on Seed Germination

**Figure 1:**
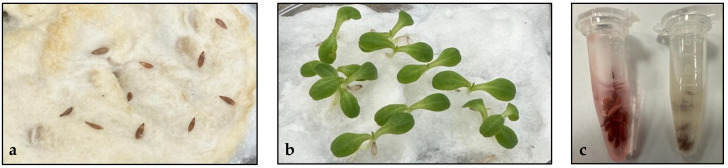
Effect of *E. coli* treatment on lettuce seed germination and embryo viability. (**a**) *E. coli*-treated lettuce seeds; (**b**) control lettuce seeds; (**c**) Tetrazolium test to check the viability of the lettuce seeds’ embryos. The Eppendorf on the right contains the *E. coli*-treated seeds, whereas the Eppendorf on the left contains the control lettuce seeds.

During the planting of the D5 plot, it was observed that no germination occurred over two consecutive tests when fields were planted using seeds. Alternatively, when lettuce plantlets were used, lettuce plants grew normally despite the *E. coli* in the soil. The in vitro analysis showed that no germination occurred when seeds were planted on cotton inoculated with *E. coli*, whereas 98% of the seeds treated with sterile water germinated (Figure 1a,b). Moreover, the ungerminated seeds were proven dead after the treatment with Tetrazolium (Figure 1c). Our results showed that the seed-germination process is highly sensitive to *E. coli* contamination and supported similar observations [70,71].

### 2.4. Effect of HMs on Bacterial Growth

Following the quantification of contaminants, it was important to understand the interaction between chemicals and bacteria coexisting in the environment. To that end, nine isolates were cultured in different concentrations of four HMs, and the isolates’ behavior was assessed over 24 h (Supplementary Figure S1A–D). When *E. coli* was not affected by the presence of Mn at all concentrations during the 24-h treatment, Zn only delayed the lag phase of the nine isolates at 100 ppm. However, Cu and Ni at 70 and 100 ppm completely inhibited the growth of the nine *E. coli* isolates. HMs seemed to affect bacterial growth differently. Mn is considered an enzyme cofactor when iron is deficient in the environment. However, it becomes toxic at high concentrations [72]. The *E. coli* genome encodes for proteins responsible for maintaining Mn’s homeostasis inside the bacterial cell. It involves Mn-dependent transcription regulators, transporters, and efflux pumps [73]. This would explain why the nine isolates tested in this study were not affected negatively by Mn. As for Zn, it was demonstrated that Zn affects the expression of the *E. coli* virulence genes rather than inhibiting its growth [74,75,76,77] while enhancing the intestine-beneficial microorganisms [78] through the antagonistic effect between beneficial and pathogenic bacteria. On the other hand, the growth of *E. coli* was not affected by the presence of Cu and Ni until it reached 70 ppm and above. Cu is a vital co-factor for certain microbial enzymes while being a potential growth-pathogen-inhibitor within living organisms [79]. Notably, the minimal concentration needed to inhibit *E. coli* in our study was much lower than that reported in other published articles. It was 250 ppm in the study conducted by [80] and 960 ppm in [81], while, in our study, 70 ppm was enough to control bacterial growth. As for Ni, it was demonstrated that this metal alters the DNA of *E. coli* and stresses it by inhibiting iron uptake [82,83]. However, a study showed that 350 ppm was needed to inhibit *E. coli* growth [84]. In this study, 70 ppm affected the isolates’ growth. It is important to mention that the detected levels of Cu and Ni in the tested samples (water, soil, and lettuce) were below the minimal concentration needed to affect bacterial growth (the maximal level of Ni found was 1.73 ppm, and the maximum level of Cu found was 12.1 ppm).

### 2.5. Testing the Effect of Irrigation Systems on E. coli Contamination

**Figure 2:**
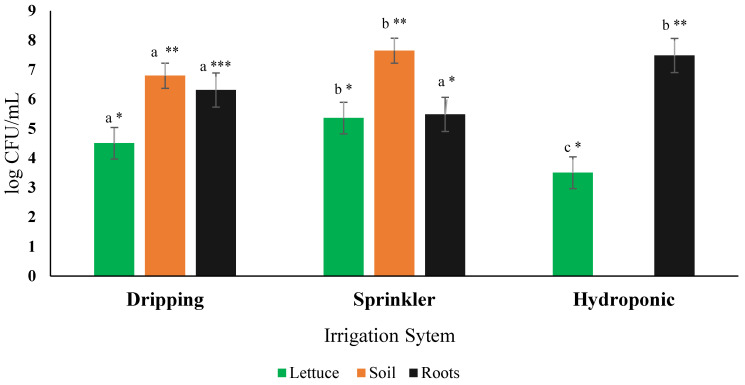
Effect of irrigation systems on *E. coli* contamination. The letters indicate the significant difference of each sample in the different irrigation systems. The asterisks (*, ** or ***) indicate the significant difference between the samples of the same irrigation system. The systems having the same number of asterisks are considered to have non-significant difference. *p*-values < 0.05.

Agricultural water has been defined as a major risk factor in contaminating leafy crops eaten raw as salads [85]. In contrast, other studies have demonstrated that bacteria were unlikely to internalize the root and contaminate the leaves via the subsurface-contaminated irrigation water [86,87]. To better understand the relationship between the irrigation water and the bacterial contamination of the romaine lettuce, three irrigation systems were tested using contaminated water: the sprinkler, the drip, and the hydroponic systems. The results showed a significant difference in *E. coli* concentrations between the three irrigation systems (Figure 2). The soil samples of the sprinklers were significantly contaminated compared to the dripping, which harbored 0.85 Logs less contamination than the sprinkler. This could be explained by the fact that the water provided by the sprinkler system will spread to the cultivated area by showering the crops and the soil when the dripping system deposits droplets of water only above the plants’ roots. Regarding the roots, the samples from the hydroponic system had the highest contamination levels (1.18 Logs more than the dripping system and 2 Logs more than the sprinkler system). At the same time, the other two systems showed no significant difference. This may be explained by the fact that the roots in the hydroponic system are immersed in the contaminated water; however, in the two other systems, the roots are attached to the soil where the bacterial cells are attached, thus preventing them from being absorbed by the roots. As for the leaves, the sprinkler also exhibited the highest level of contamination (5.36 Logs), followed by the dripping system (4.51 Logs), and, finally, the hydroponic system (3.51 Logs). This is normal because the water is directly applied to the leaves in the case of the sprinkler system. It was also demonstrated that *E. coli* can enter the leaves via the stomata [88,89]. When comparing the dripping system to the hydroponic system, *E. coli* was also detected in the leaves but in significantly higher concentrations in the dripping system (90% (1 Log) more). The presence of *E. coli* in the leaves from the dripping and hydroponic systems is in accordance with the studies that demonstrated the internalization of *E. coli* [90,91]. However, the differences in the rate of internalization through the roots between the dripping system and the hydroponic system led to questions regarding the mechanism that decreases the rate of *E. coli* translocation from the roots to the leaves.

Taken together, these results illustrate the contamination pathway for *E. coli.* Direct deposition of *E. coli* onto roots in the hydroponic system led to the highest contamination levels on the roots. However, there is little direct contact between the contaminated water and lettuce leaves in the hydroponics system, leading to low levels of contamination in the edible portions of the plant, making it the safest option for human consumption. The highest levels of *E. coli* contamination were observed within the sprinkler system in the soil. However, unlike in the hydroponics system, contaminated water is transferred directly onto both the soil and leaves, leading to high levels of *E. coli* contamination in lettuce leaves, making it the least safe system for human consumption. This higher risk was reflected in the field trials described above and the widespread *E. coli* contamination observed in D5, which used sprinkler irrigation. Between these three types of irrigation systems, drip irrigation was intermediate between these two systems, with lower levels of *E. coli* contamination in lettuce compared to sprinkler irrigation but higher levels of *E. coli* contamination in lettuce compared to hydroponics systems. Again, this was also reflected in our field trials, which showed mixed results regarding the safety of drip irrigation in the context of contaminated water. In D3, contaminated water did not lead to contaminated soils or lettuce, whereas in D1 and D2, soils and lettuce remained uncontaminated.

Due to the high levels of nutrients and organic matter in the soil, in addition to the warm temperatures, *E. coli* levels can survive in the soil for a long period, establishing a commensal relationship in which, in the absence of predation and competition, it maintains stable populations in soil [41,92]. These warm soil temperatures may prolong contamination, making remediation challenging following the original contamination event. Warming temperatures lead to high levels of *E. coli* contamination in soils, presenting a specific threat to sites that cultivate lettuce in the summer/fall and utilizing sprinkler irrigation.

### 2.6. Effect of High Levels of Nickel on E. coli Survival in the Environment

Nickel was added to the contaminated irrigation water at 70 mg/L following the completion of the irrigation experiment described in Section 4 to understand how HMs impact *E. coli* contamination. While higher than the levels of Ni observed in irrigation water during this experiment (<2 μg/L), this level was chosen instead to investigate if excessive levels of HM contamination would limit *E. coli* contamination and, if so, which system would be the most impacted.

**Figure 3:**
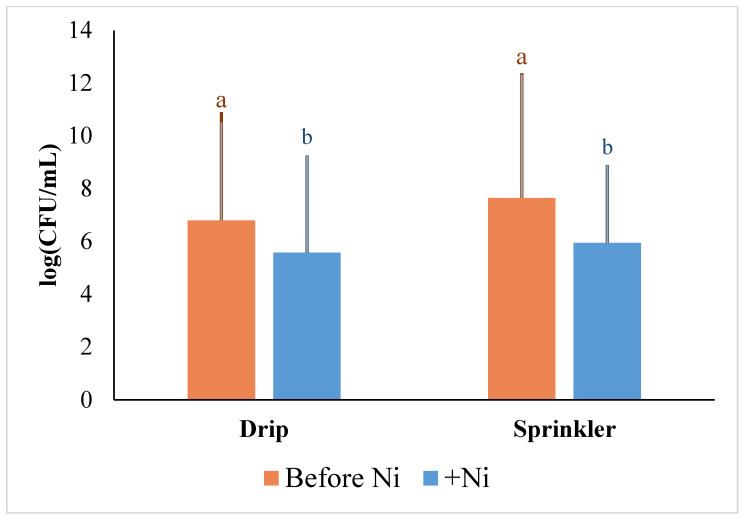
Impact of Ni treatment on *E. coli* contamination in soil in drip and sprinkler irrigation systems. Letters show statistically significant differences identified using paired *t*-tests on data from each irrigation system. *p*-value < 0.05.

**Figure 4:**
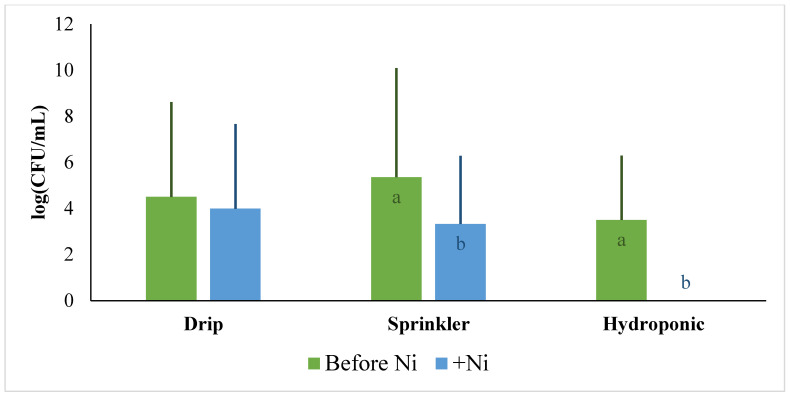
Impact of Ni treatment on *E. coli* lettuce contamination in drip, sprinkler, and hydroponic irrigation systems. Letters show statistically significant differences identified using paired *t*-tests on data from each irrigation system. *p*-value < 0.05.

Within both sprinkler and drip systems, *E. coli* contamination of the soil was significantly reduced after Ni treatment (Figure 3). However, Ni addition did not reduce *E. coli* contamination of lettuce leaves under drip irrigation (Figure 4). The lack of response within lettuce leaves is likely due to Ni being primarily deposited and retained in the soil under drip irrigation. Soils can retail much greater concentrations of HM through various mechanisms, such as chelation [93], thus preventing metals, such as Ni, from reaching *E. coli*, which is already present in or on lettuce leaves [94]. Alternatively, under sprinkler irrigation, more Ni is likely directly deposited onto plant leaves, bringing *E. coli* directly into contact with elevated concentrations of Ni. This was reflected in the significant decrease in *E. coli* in lettuce leaves following Ni treatment (Figure 4).

Under hydroponic irrigation, the lettuce leaf contamination with *E. coli* dropped to below 10 CFU/mL, resulting in the approximate elimination of bacteria (Figure 4). This result can be explained by the high phyto-availability of soluble Ni in the soilless system, as dissolved elements are much more bioavailable to plants than soil-associated elements, thus allowing for maximal accumulation of Ni within plant tissue, which comes into contact with *E coli*. Additionally, without soil, *E. coli* in the irrigation water is continuously exposed to excessive Ni concentrations, thus reducing further contamination.

The findings of this experiment underscore the effectiveness of targeted approaches in mitigating *E. coli* contamination in agricultural systems. While Ni demonstrated significant toxicity towards *E. coli* and led to a noticeable reduction in contamination levels, Ni may have negative plant or human health consequences at excessively high concentrations. The recommendation of this study is not to promote the use of Ni as a solution for contamination but rather to illustrate the possible competition between differing sources of contamination. These results emphasize the importance of careful consideration and further research into the potential environmental impacts and safety implications of using HMs like Ni in agricultural practices.

## 3. Materials and Methods

### 3.1. Demonstration Plot Selection and Sample Collection

**Figure 5:**
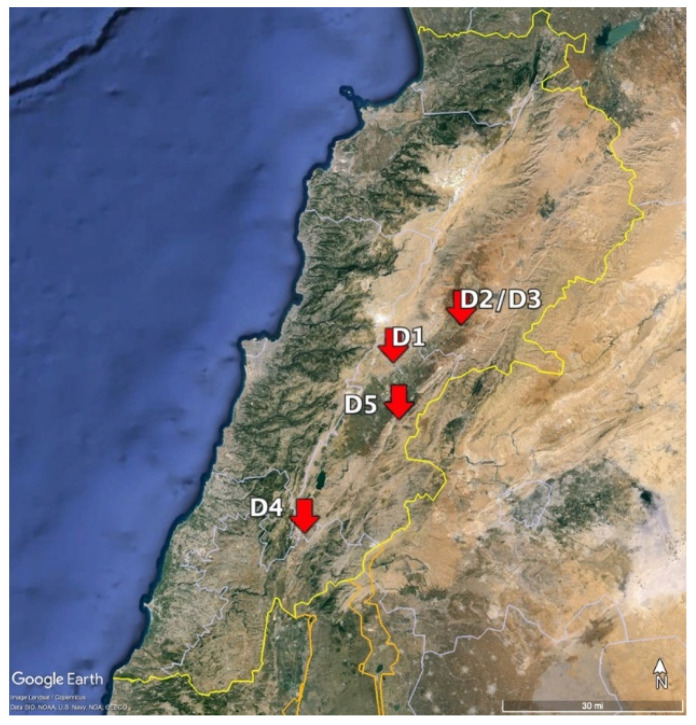
Map depicting the five demonstration plots (D1–5).

Five demonstration plots were chosen to conduct this study. Different criteria were adopted for the selection. The plots were strategically located around the LRB to assess the impact of the local river water’s quality on crop production, especially in areas known for heavy agricultural activity. The chosen plots represent a variety of agricultural practices used by key lettuce farmers in the region. The selection also includes plots operated by prominent lettuce growers to reflect the practices of those with significant influence in the industry. Additionally, the plots were chosen to cover different seasonal growth patterns, thus allowing for observing how various agricultural practices and environmental conditions affect lettuce production throughout the year (Table 3, Figure 5). This diversity in plot selection ensures that the study results will be widely applicable to the agricultural community around the Litani River.

Each plot was divided into two sections acting as replicas. In the plots where greenhouses were used, each replica consisted of one greenhouse, except for D4, where two greenhouses were placed in each part. Therefore, G1–G2 were in the first replica, and G3–G4 were in the second replica. Before the planting season, information about agricultural practices was collected to understand better farmers’ adopted agricultural practices and to identify gaps and malpractices.

### 3.2. Microbiological and Chemical Analysis

Water and soil samples were collected from each plot before planting and subjected to bacterial and heavy metal quantification, as described below. Lettuce, water, and soil samples were collected during the final season. In sterilized one-liter bottles, 2 L of irrigation water was collected directly from the water source. For lettuce samples, composite leaf samples formed from different lettuce plants were gathered, alternating between inner and outer leaves, and placed in sterile bags. In the case of soil samples, a 1 kg composite sample was prepared and placed in sterile plastic bags. All collected samples were immediately placed on ice and transported to the Food Microbiology Laboratory at the Faculty of Agricultural and Food Sciences labs at the American University of Beirut, where appropriate testing was conducted to quantify the presence of bacteria and heavy metals.

#### 3.2.1. *E. coli* and *Salmonella* Detection

To detect *E. coli* in the water samples, 1 L of water was filtered using Whatman filter paper. The filter was then placed on Rapid *E. coli* 2 agar (Bio-rad, Hercules, CA, USA) and incubated at 37 °C for 24 h.

For *E. coli* detection in lettuce samples, leaves were cut using a sterile knife and a sterile cutting board. Five samples were then prepared by weighing 25 g of lettuce in a stomacher bag and adding 225 mL of Buffered Peptone Water (BPW) (Himedia, Thane, India). The samples were then blended using a stomacher and incubated at 37 °C for 24 h. Serial dilutions were prepared, and the samples were plated on Rapid *E. coli* 2 agar, followed by incubation at 37 °C for 24 h.

For the soil samples, 25 g of soil was weighed in a stomacher bag, and 225 mL of BPW was added. The mixture was then blended using a stomacher and incubated at 37 °C for 24 h. Serial dilution was performed, and the samples were plated on Rapid *E. coli* 2 agar, followed by incubation at 37 °C for 24 h. Suspected colonies were confirmed using Api20E strips (Biomerieux, Craponne, France). MacConkey agar (Himedia, Thane, India) was utilized for the purification process, and the isolates were subsequently stored in 80% glycerol stocks at −80 °C for further investigation. All tests were performed in duplicate.

The following process was adopted to detect *Salmonella* sp. For water samples, 1 L was filtered using Whatman filter paper to capture bacterial cells. The filter paper was placed in a 9 mL falcon tube containing BPW and incubated at 37 °C for 24 h. For soil and lettuce samples, the pre-prepared mixtures (25 g of the sample + 225 mL of BPW) were incubated at 37 °C for 24 h. The following day, 1 mL of the incubated BPW was transferred to a 9 mL Rappaport-Vassiliadis *Salmonella* (RVS) enrichment medium (Himedia, Thane, India) and further incubated at 41.5 °C for 24 h. After the second incubation, a full loop of the RVS culture was streaked on Xylose Lysine Deoxycholate agar (XLD agar) and *Salmonella-Shigella* agar (SS agar) (Himedia, Thane, India). Suspected colonies were confirmed using the *Salmonella* Latex Test (Bio-rad, Hercules, CA, USA) and Api20E tests. The tests were performed in duplicate.

#### 3.2.2. Heavy Metal Quantification

Water samples were acidified to 1% nitric acid (*v*/*v*) and kept at 4 °C until analysis. Soils were first air-dried at room temperature for at least one week (length of drying dependent on the moisture of the soil), ground to a fine powder using a mortar and pestle, and then subjected to a DTPA (Diethylenetriaminepentaacetic acid) extraction following standard methods to analyze bioavailable metals [95]. Lettuce samples used for heavy metal analysis were oven-dried for 48 h at 55 °C, ground to a fine powder, and then subjected to microwave digestion. For this digestion, 8 mL of HNO_3_ and 2 mL of H_2_O_2_ were added to 0.4 g of lettuce samples in the Teflon digestion vessels. Samples were digested at 200 °C (25 min ramp time, 15 min hold time). Quantification of Ni and Cu in all sample types was conducted using graphite furnace atomic absorption spectrometry (GF-AAS, Thermo Scientific iCE 3000 Series, Waltham, MA, USA). Meanwhile, Zn analysis for all sample types was performed via flame atomic absorption spectrometry (FAAS). Soil analysis of Mn was carried out using FAAS, whereas Mn quantification of lettuce and water samples was performed using GF-AAS.

### 3.3. Effect of E. coli on Seed Germination

To assess the impact of *E. coli* contamination on germination, lettuce seeds were immersed in distilled water for 24 h to initiate germination. Subsequently, the seeds were divided into two groups. The control seeds were placed on water-soaked cotton in a sterile Petri dish, and the *E. coli*-treated group was immersed in *E. coli*-soaked cotton for 10 days. The embryos within the seeds were judged as viable if red appeared after submerging them in a Tetrazolium (Himedia) solution for 24–48 h.

### 3.4. Effect of HMs on Bacterial Growth

Because *E. coli* and HM co-existed in the environment, the effect of the HMs on the isolate behavior was studied in vitro. To do that, nine isolates were selected—three from water (W1–3), lettuce (L1–3), and soil (S1–3) samples—and mixed with different concentrations of four HMs (Cu, Ni, Zn, and Mn). Optical Density (OD) at 600 nm was measured and compared to the control over a period of 24 h.

#### 3.4.1. Preparation of the Isolates

The isolates were revived from the stored stocks through streaking on Rapid *E. coli* 2 agar and incubated overnight at 37 °C. Two to three colonies were used for each isolate to inoculate 1 L of BPW and incubated at 37 °C with constant shaking to reach a concentration of 2 MacFarland Units (MFU), corresponding to an OD_600_ of 0.2.

#### 3.4.2. Preparation of the HM Solutions

Four HM salts (copper II sulfate ((CuSO_4_·5H_2_O), MERCK, Rahway, NJ, USA), nickel sulfate ((NiSO_4_), UNI-CHEM, Greenville, SC, USA), zinc sulfate (ZnSO_4_·7H_2_O), MERCK, Rahway, NJ, USA), and manganese sulfate ((MnSO_4_·H_2_O), FLUKA, Buchs, Switzerland) were used to prepare 500 mL stock solutions of 1000 ppm using sterile distilled water. The amount of metal salt used was calculated using the following equation:

$$amount\;of\;metal\;salt=\frac{molecular\;weight\;of\;metal\;salt}{molar\;mass\;of\;target\;metal}$$

From the initial 1000 ppm HM stock, a serial dilution using BPW was prepared. Seven treatments were prepared for each HM containing 2, 10, 20, 40, 100, 140, and 200 ppm of the metal.

#### 3.4.3. Preparation of the Solutions Containing HMs and *E. coli*

To prepare the solutions containing *E. coli* and HMs, 10 mL of the bacterial solution was added to 10 mL of the HM solution. This resulted in seven solutions containing 1 MFU (OD_600_ of 0.1) of *E. coli* and HMs at 1, 5, 10, 20, 50, 70, and 100 ppm, respectively. Additionally, a control was prepared by mixing 10 mL of the bacterial solution with 10 mL of BPW without HMs in a 50 mL conical tube for comparison. Also, a blank solution of only HMs in BPW served as a second control. The solutions were incubated at 37 °C with constant shaking. The OD_600_ was measured at five time points (0 h, 5 h, 10 h, 15 h, and 24 h), and viability was confirmed using MacConkey agar. The (OD_600_ vs. time) curve of the isolates at each concentration was then plotted and compared to the control.

### 3.5. Effect of the Irrigation Systems on Bacterial Contamination

After evaluating the behavior of the *E. coli* isolated from the environment in the presence of HMs, it was necessary to evaluate the route of *E. coli* into the lettuce leaves and suggest the best system to mitigate bacterial contamination. A total of 120 romaine lettuce plantlets were purchased from a local nursery, planted in compost soil, and grown under three different irrigation systems, drip, sprinkler, and hydroponic, at 24 °C at the AUB-FAFS greenhouses. The irrigation water (bacteria-free drinking water) used for those three systems was inoculated with *E. coli*. A control group for each system was applied using non-inoculated water.

#### 3.5.1. Preparation of the Irrigation Water

One of the water isolates was selected for this part. From the stock stored at −80 °C, a full loop was taken and streaked on Rapid *E. coli* 2 agar, followed by incubation at 37 °C overnight. Two to three colonies were then used to inoculate 1400 mL of BPW and incubated at 37 °C with constant shaking overnight. To prepare the contaminated irrigation water, BPW containing *E. coli* was diluted using *E. coli*-free drinking water to reach a concentration of 3 MFU, corresponding to the most active bacterial-growth phase. This concentration was chosen based on the level of contamination found in the irrigation water tested.

#### 3.5.2. Comparison between Irrigation Systems

Lettuce and soil samples from the three different irrigation systems were collected after 45 days, and the same microbiological testing methodology described above was employed for analysis. As no soil was used in the hydroponic system, only water was tested by utilizing the same testing protocol described above, except the water was diluted tenfold using autoclaved, distilled water before filtration. In addition, samples of roots from each system were collected. The roots were washed to remove any soil by soaking them in distilled water and then sterilized using 10% sodium hypochlorite. Afterward, 25 g of each root sample was weighed in a stomacher bag, and 225 mL of BPW was added. The samples were then blended using a stomacher, serially dilated, plated on Rapid *E. coli* 2 agar, and incubated for 24 h at 37 °C. The results were compared to the corresponding control. These tests were performed in duplicate.

#### 3.5.3. Effect of Nickel on *E. coli*’s Presence

Following the above experiment, a group of *E. coli*-treated samples was subjected to further treatment with 70 mg/L of Ni, representing the Minimum Inhibitory Concentration (MIC) for the bacterium tested in a previous experiment. The Ni solution was prepared by dissolving nickel sulfate (NiSO_4_) salt in autoclaved, distilled water to prepare 500 mL stock solutions of 1000 mg/L. Then, the stock was diluted using sterile drinking water to obtain a concentration of 70 mg/L of Ni. This treatment was applied to all three irrigation systems, and samples from the soil (sprinklers and dripping system), water (hydroponic system), and lettuce (all) in each system were tested for the presence of *E. coli* using the same method described earlier.

### 3.6. Statistical Analysis

Statistical analysis was undertaken using R studio software (R.4.3.0). Initially, data were tested for non-normality using the Shapiro–Wilk test. Datasets that did not fail the Shapiro–Wilk test (i.e., assumed normal) were analyzed using one-way ANOVA followed by Tukey’s Honestly Significant Difference (Tukey HSD) test. The Kruskal–Wallis test was conducted on data not normally distributed even after data transformation, followed by Dunn’s test to assess pairwise differences across groups. To evaluate the differences in bacterial contamination among the irrigation systems, a paired *t*-test was used when data were normally distributed, and the Wilcoxon signed-rank test was used when the normality test was rejected despite data transformation. *p*-values of less than 0.05 were used to determine the statistical significance of differences between groups.

## 4. Conclusions

This study expanded the scientific and public health communities’ understanding of the intricate dynamics of *E. coli* contamination and HMs in agricultural environments, particularly in Lebanon’s vulnerable LRB. By shedding light on the seasonal variations, sources of contamination (e.g., soil vs. water), and potential adaptation of *E. coli* in the environment, we hope to provide fundamental knowledge to support innovative strategies to mitigate risks to human health and environmental sustainability. Our work also underscores the urgency of adopting responsible agricultural practices and sanitary water-management systems to address the challenges posed by *E. coli*, thus offering new perspectives for future research, practical interventions, and transformative approaches that safeguard food safety and promote ecological resilience in agricultural landscapes. This study reveals a significant correlation between *E. coli* contamination and the presence of HM in the LRB. Key findings include the identification of hotspots where both contaminants co-occur at alarming levels, suggesting potential health risks and environmental impacts. The research also highlights the role of agricultural runoff as a primary source of these contaminants, providing valuable insights for targeted intervention strategies. These contributions underscore the urgent need for improved monitoring and management practices in the LRB. This paper addresses farmers, policymakers, and concerned stakeholders, such as ministries, as it is paramount to draw up a strict work plan to resolve environmental contamination at its source. Many strategies could be implemented, such as creating an improved waste-management system, adopting sustainable farming practices, and establishing regular monitoring and community engagement.

## Figures and Tables

**Table 1:** Microbiological screening of soil, water, and lettuce in the five demonstration plots during the pre-planting and post-harvest seasons. +: presence of *E. coli*; −: absence of *E. coli*. (D) refers to the demonstration plot, and (G) refers to the greenhouse. Experiments were run in duplicate on two plots, and the results below were consistent between both plots.

	Water		Soil		Lettuce
Demo Plot	Pre-Planting	Harvest	Pre-Planting	Harvest	Harvest
D1	+	−	−	−	−
D2	+	+	−	−	−
D3	+	−	−	+	+
D4	−	+	− (G1/G3)	− (G1/G3)	− (G1/G3)
			+ (G2/G4)	+ (G2/G4)	+ (G2/G4)
D5	−	+	+	+	+

**Table 2:** Quantification of nickel, zinc, copper, and manganese available in lettuce and soil (ppm). Statistical groupings from post hoc tests (Tukey or Dunn’s test) are shown with lettered superscripts. Values sharing the same letter are not significantly different. Standard error values are given following ± symbol.

Time	Sample	Location	Ni	Zn	Cu	Mn
Pre-planting	Soil	D1	0.89 ± 0.02 ^a^	2.05 ± 0.02 ^a^	0.94 ± 0.06 ^b^	12.4 ± 0.4 ^b^
		D2	1.26 ± 0.09 ^a^	2.82 ± 0.98 ^a^	1.66 ± 0.22 ^ab^	144 ± 15 ^a^
		D3	1.29 ± 0.08 ^a^	2.90 ± 0.59 ^a^	1.62 ± 0.15 ^ab^	135.6 ± 9.5 ^a^
		D4	0.42 ± 0.04 ^b^	0.80 ± 0.08 ^b^	1.33 ± 0.10 ^b^	7.5 ± 0.7 ^c^
		D5	0.32 ± 0.03 ^b^	2.32 ± 0.41 ^a^	1.93 ± 0.14 ^a^	11.6 ± 0.8 ^b^
Final	Soil	D1	0.98 ± 0.03 ^a^	1.99 ± 0.55 ^b^	1.05 ± 0.04 ^c^	12.7 ± 0.7 ^b^
		D2	1.14 ± 0.20 ^a^	3.99 ± 0.68 ^a^	1.90 ± 0.16 ^a^	100 ± 18 ^a^
		D3	0.96 ± 0.17 ^a^	3.49 ± 0.36 ^a^	1.88 ± 0.25 ^a^	116 ± 8 ^a^
		D4	0.40 ± 0.04 ^b^	0.78 ± 0.05 ^c^	1.36 ± 0.08 ^bc^	7.8 ± 1.0 ^c^
		D5	0.27 ± 0.02 ^b^	1.81 ± 0.33 ^b^	1.74 ± 0.16 ^ab^	5.3 ± 0.5 ^c^
	Lettuce	D1	1.16 ± 0.07 ^b^	16.7 ± 1.6 ^d^	12.1 ± 2.4 ^a^	61.7 ± 7.2 ^c^
		D2	0.54 ± 0.03 ^c^	170 ± 11 ^a^	10.4 ± 2.6 ^b^	118.0 ± 4.9 ^b^
		D3	1.08 ± 0.20 ^b^	23.7 ± 5.9 ^c^	8.36 ± 0.66 ^b^	107.5 ± 7.9 ^b^
		D4	1.33 ± 0.27 ^ab^	45.2 ± 2.9 ^b^	10.3 ± 0.36 ^a^	163 ± 10 ^a^
		D5	1.73 ± 0.34 ^a^	25.5 ± 2.2 ^c^	7.57 ± 0.55 ^b^	112.6 ± 9.9 ^b^

**Table 3:** Geographical locations and details about the demonstration plots.

Plot ID	Location	GPS Location	Setup	Irrigation Type	Growing Season	Lettuce Planted: Seeds or Seedlings
D1	Zahle	33.84675, 35.90203	Open field	Drip	October 2022–January 2023	Seedlings
D2	Talya	33.9366930, 36.0968463	Greenhouse	Drip	October 2022–January 2023	Seedlings
D3	Talya	33.9366930, 36.0968463	Greenhouse	Drip	December 2022–March 2023	Seedlings
D4	Lousse	33.441124, 35.6493756	Greenhouse	Drip	July–September 2023	Seedlings
D5	Majdal Anjar	33.7123210, 35.9182159	Open field	Sprinkler	August–November 2023	Seeds

## Data Availability

The original contributions presented in the study are included in the article/Supplementary Materials; further inquiries can be directed to the corresponding author.

## Supplementary Materials

The following supporting information can be downloaded at https://www.mdpi.com/article/10.3390/plants13172460/s1, Figure S1. The behavior of the *E. coli* isolated from water (W1, W2, W3), lettuce (L1, L2, L3), and soil (S1, S2, S3) treated with different concentrations of (A) Cu, (B) Mn, (C) Zn, and (D) Ni over a period of 24 h; Table S1. Biological Absorption Coefficient (BAC) in lettuce leaves, pH, and Soil Organic Matter (SOM) of soil in the five demo plots at the harvesting stage.
