# Supplementary material for: Agricultural Mitigation Strategies to Reduce the Impact of Romaine Lettuce Contamination
Source: Plants (Basel). 2024 Sep 3;13(17):2460. doi: 10.3390/plants13172460 (PMC11396837; doi:10.3390/plants13172460)
Supplement: Supplementary file 1 [file plants-13-02460-s001.zip › Figure S1.pptx]

## Slide 1
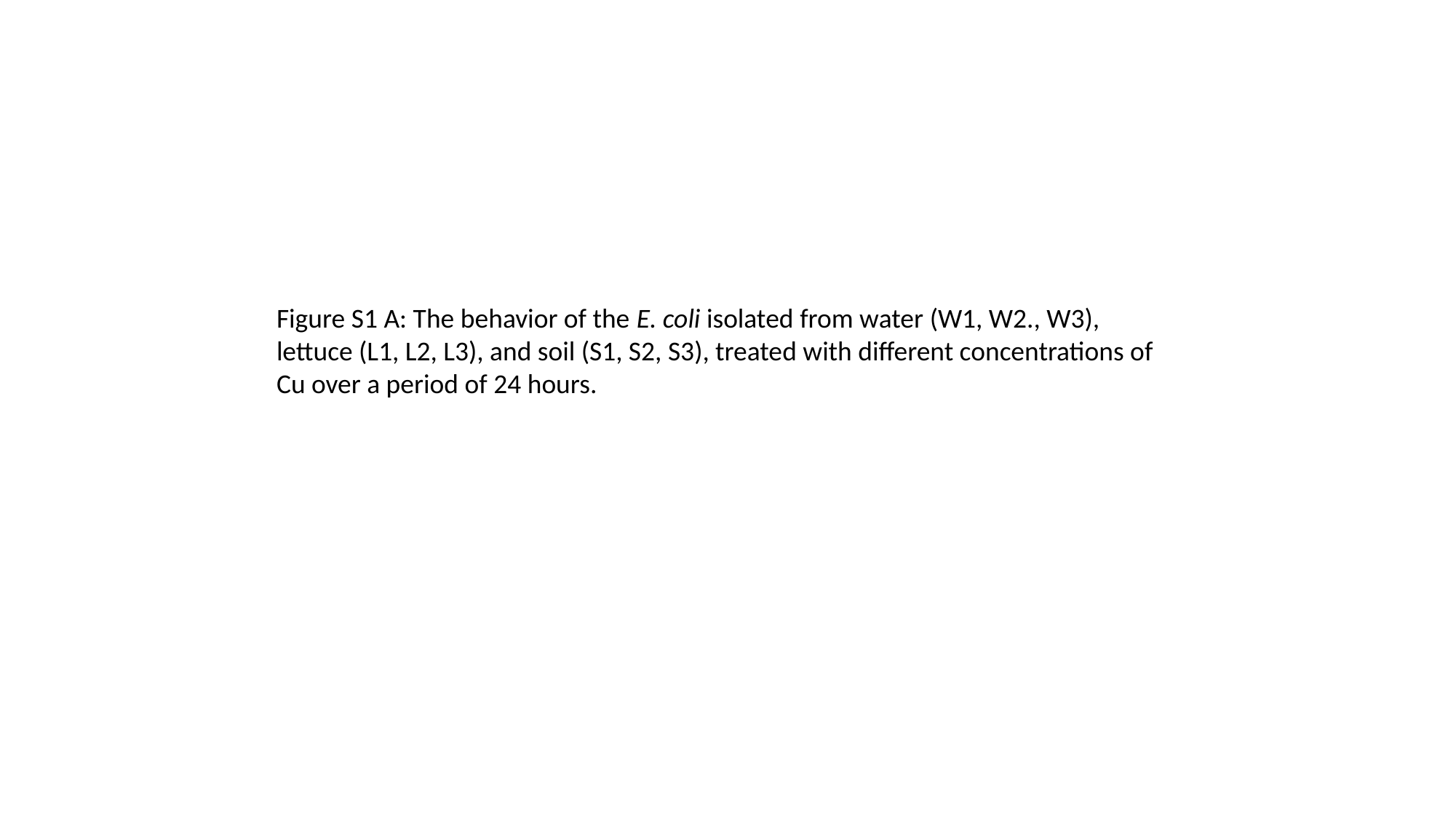

Figure S1 A: The behavior of the E. coli isolated from water (W1, W2., W3), lettuce (L1, L2, L3), and soil (S1, S2, S3), treated with different concentrations of Cu over a period of 24 hours.

## Slide 2
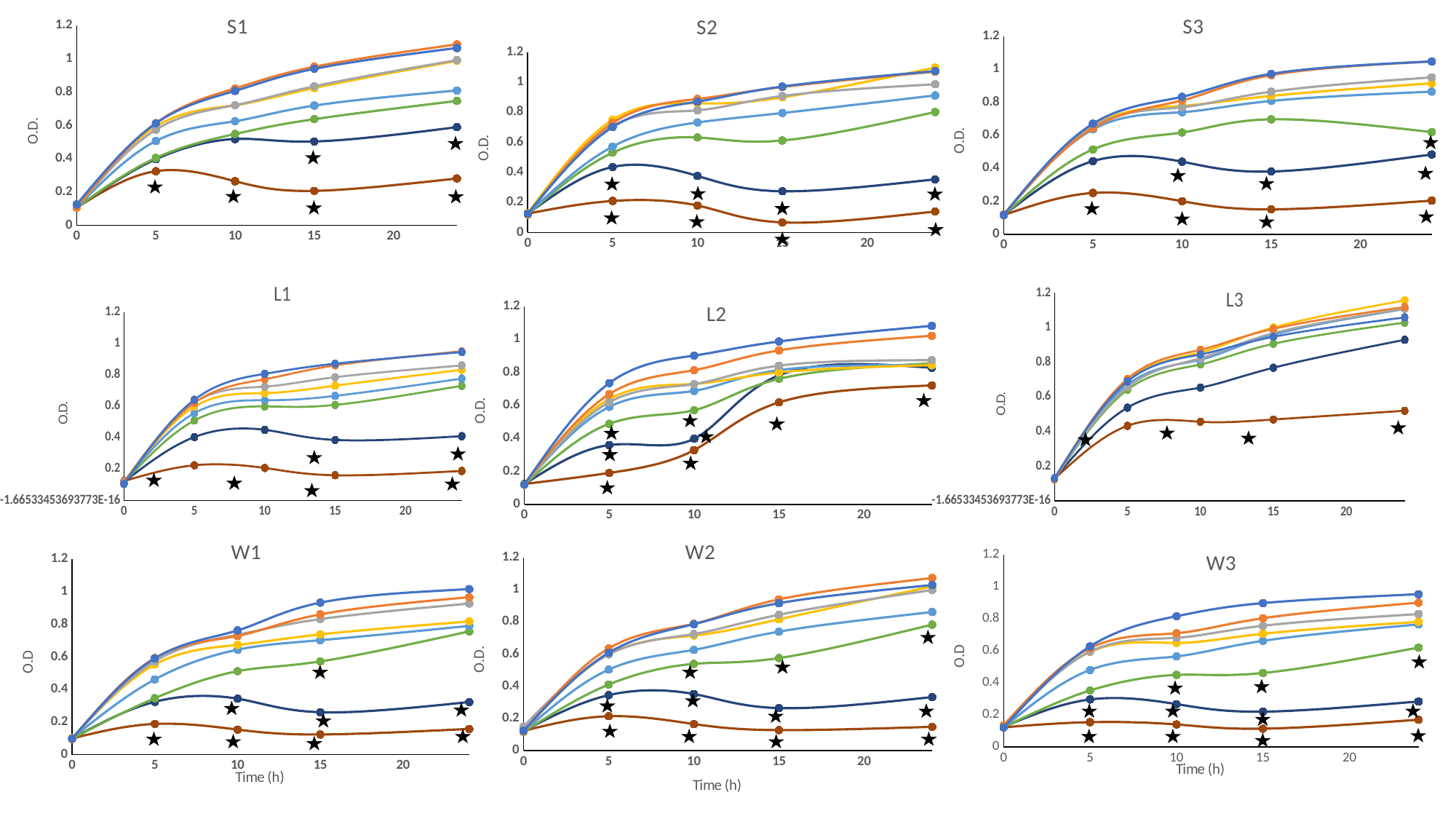

### Chart: S2
| Category | control | 1ppm | 5ppm | 10ppm | 20ppm | 50ppm | 70ppm | 100ppm |
|---|---|---|---|---|---|---|---|---|
### Chart: S3
| Category | control | 1ppm | 5ppm | 10ppm | 20ppm | 50ppm | 70ppm | 100ppm |
|---|---|---|---|---|---|---|---|---|
### Chart: S1
| Category | control | 1ppm | 5ppm | 10ppm | 20ppm | 50ppm | 70ppm | 100ppm |
|---|---|---|---|---|---|---|---|---|
### Chart: L1
| Category | control | 1ppm | 5ppm | 10ppm | 20ppm | 50ppm | 70ppm | 100ppm |
|---|---|---|---|---|---|---|---|---|
### Chart: L2
| Category | control | 1ppm | 5ppm | 10ppm | 20ppm | 50ppm | 70ppm | 100ppm |
|---|---|---|---|---|---|---|---|---|
### Chart: L3
| Category | control | 1ppm | 5ppm | 10ppm | 20ppm | 50ppm | 70ppm | 100ppm |
|---|---|---|---|---|---|---|---|---|
### Chart: W2
| Category | control | 1ppm | 5ppm | 10ppm | 20ppm | 50ppm | 70ppm | 100ppm |
|---|---|---|---|---|---|---|---|---|
### Chart: W3
| Category | control | 1ppm | 5ppm | 10ppm | 20ppm | 50ppm | 70ppm | 100ppm |
|---|---|---|---|---|---|---|---|---|
### Chart: W1
| Category | control | 1ppm | 5ppm | 10ppm | 20ppm | 50ppm | 70ppm | 100ppm |
|---|---|---|---|---|---|---|---|---|

## Slide 3
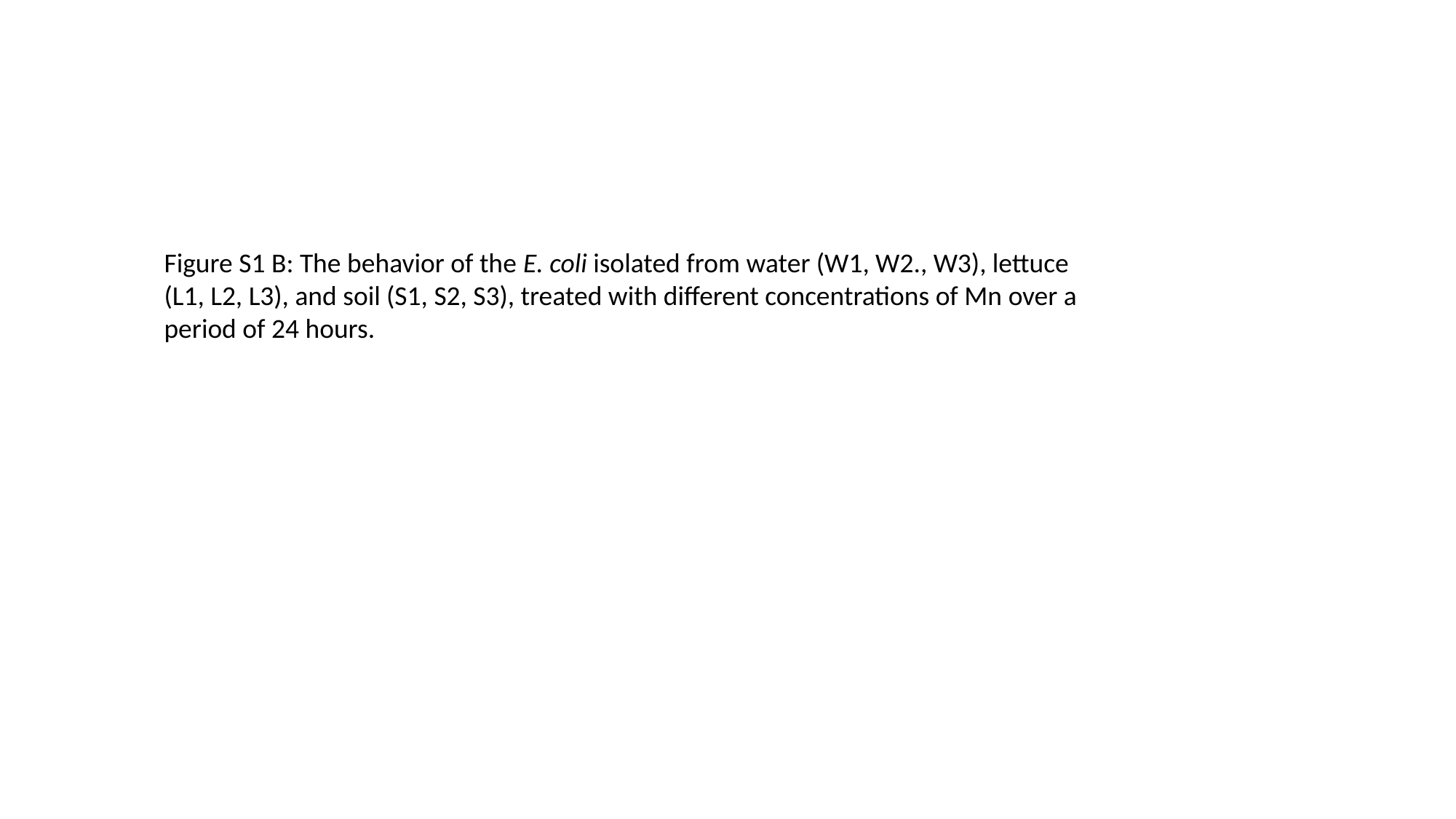

Figure S1 B: The behavior of the E. coli isolated from water (W1, W2., W3), lettuce (L1, L2, L3), and soil (S1, S2, S3), treated with different concentrations of Mn over a period of 24 hours.

## Slide 4
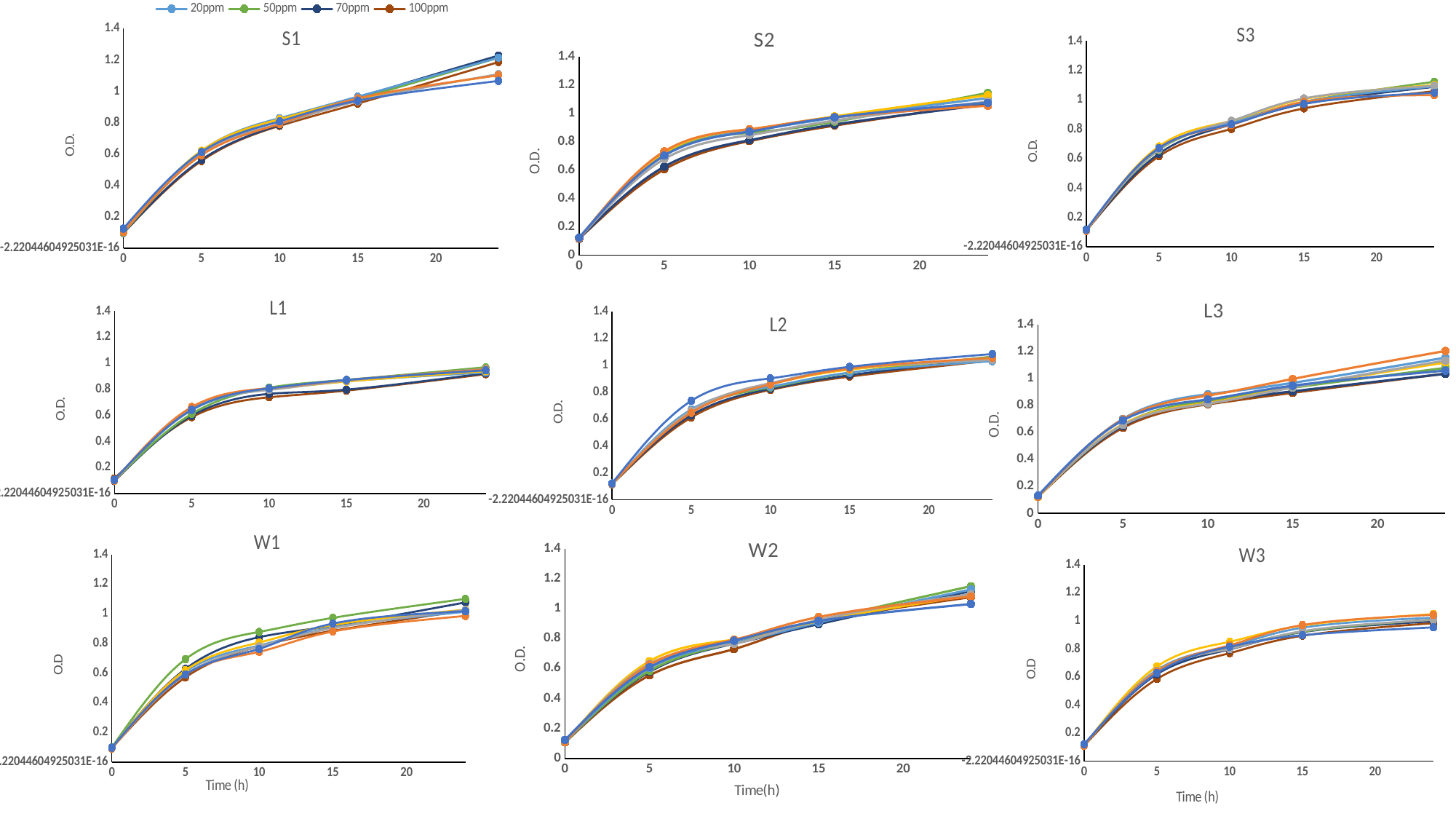

### Chart: S1
| Category | control | 1ppm | 5ppm | 10ppm | 20ppm | 50ppm | 70ppm | 100ppm |
|---|---|---|---|---|---|---|---|---|
### Chart: S3
| Category | control | 1ppm | 5ppm | 10ppm | 20ppm | 50ppm | 70ppm | 100ppm |
|---|---|---|---|---|---|---|---|---|
### Chart: S2
| Category | control | 1ppm | 5ppm | 10ppm | 20ppm | 50ppm | 70ppm | 100ppm |
|---|---|---|---|---|---|---|---|---|
### Chart: L3
| Category | control | 1ppm | 5ppm | 10ppm | 20ppm | 50ppm | 70ppm | 100ppm |
|---|---|---|---|---|---|---|---|---|
### Chart: L1
| Category | control | 1ppm | 5ppm | 10ppm | 20ppm | 50ppm | 70ppm | 100ppm |
|---|---|---|---|---|---|---|---|---|
### Chart: L2
| Category | control | 1ppm | 5ppm | 10ppm | 20ppm | 50ppm | 70ppm | 100ppm |
|---|---|---|---|---|---|---|---|---|
### Chart: W2
| Category | control | 1ppm | 5ppm | 10ppm | 20ppm | 50ppm | 70ppm | 100ppm |
|---|---|---|---|---|---|---|---|---|
### Chart: W3
| Category | control | 1ppm | 5ppm | 10ppm | 20ppm | 50ppm | 70ppm | 100ppm |
|---|---|---|---|---|---|---|---|---|
### Chart: W1
| Category | control | 1ppm | 5ppm | 10ppm | 20ppm | 50ppm | 70ppm | 100ppm |
|---|---|---|---|---|---|---|---|---|

## Slide 5
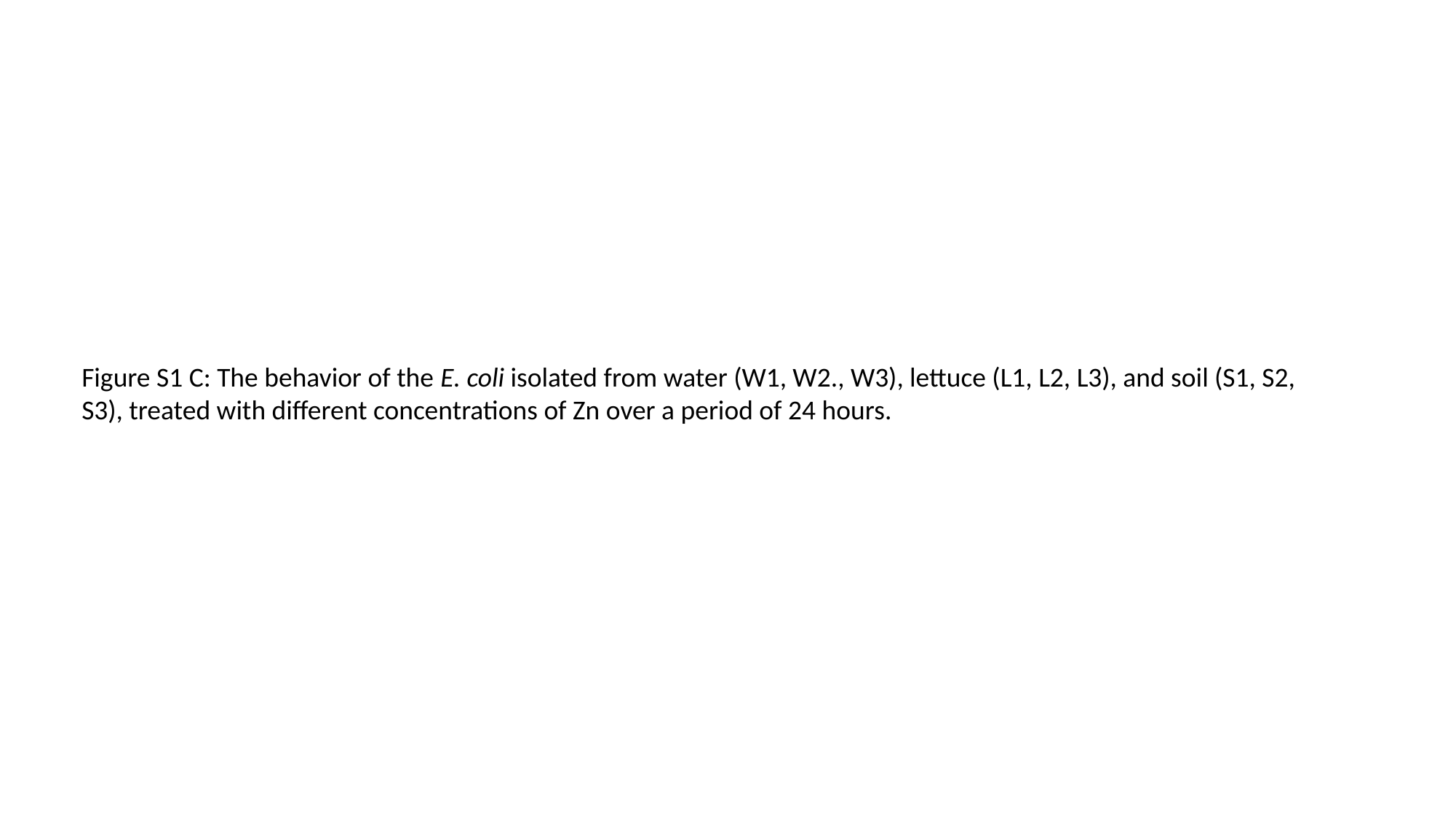

Figure S1 C: The behavior of the E. coli isolated from water (W1, W2., W3), lettuce (L1, L2, L3), and soil (S1, S2, S3), treated with different concentrations of Zn over a period of 24 hours.

## Slide 6
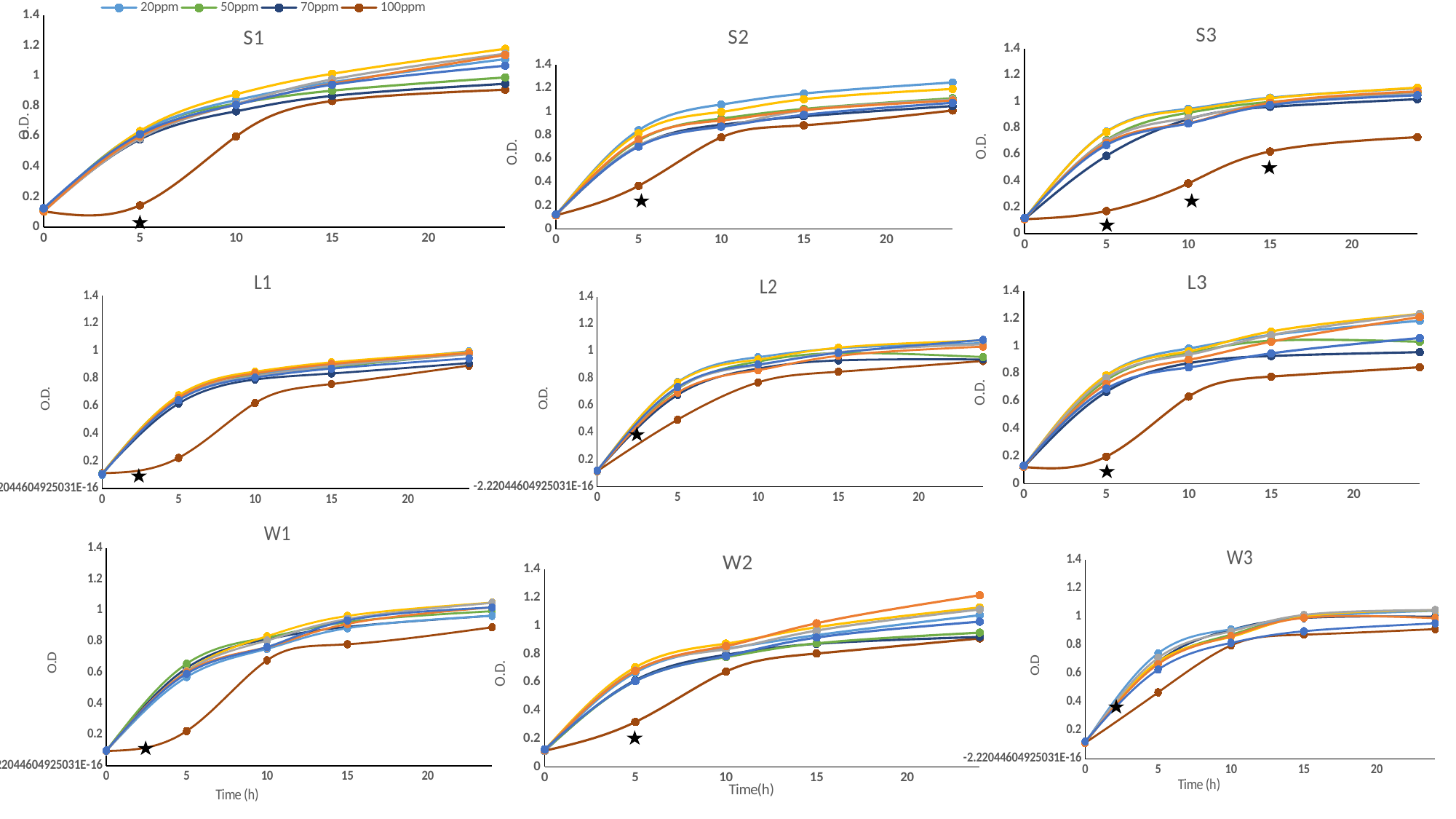

### Chart: S3
| Category | control | 1ppm | 5ppm | 10ppm | 20ppm | 50ppm | 70ppm | 100ppm |
|---|---|---|---|---|---|---|---|---|
### Chart: S1
| Category | control | 1ppm | 5ppm | 10ppm | 20ppm | 50ppm | 70ppm | 100ppm |
|---|---|---|---|---|---|---|---|---|
### Chart: S2
| Category | control | 1ppm | 5ppm | 10ppm | 20ppm | 50ppm | 70ppm | 100ppm |
|---|---|---|---|---|---|---|---|---|
### Chart: L3
| Category | control | 1ppm | 5ppm | 10ppm | 20ppm | 50ppm | 70ppm | 100ppm |
|---|---|---|---|---|---|---|---|---|
### Chart: L1
| Category | control | 1ppm | 5ppm | 10ppm | 20ppm | 50ppm | 70ppm | 100ppm |
|---|---|---|---|---|---|---|---|---|
### Chart: L2
| Category | control | 1ppm | 5ppm | 10ppm | 20ppm | 50ppm | 70ppm | 100ppm |
|---|---|---|---|---|---|---|---|---|
### Chart: W1
| Category | control | 1ppm | 5ppm | 10ppm | 20ppm | 50ppm | 70ppm | 100ppm |
|---|---|---|---|---|---|---|---|---|
### Chart: W3
| Category | control | 1ppm | 5ppm | 10ppm | 20ppm | 50ppm | 70ppm | 100ppm |
|---|---|---|---|---|---|---|---|---|
### Chart: W2
| Category | control | 1ppm | 5ppm | 10ppm | 20ppm | 50ppm | 70ppm | 100ppm |
|---|---|---|---|---|---|---|---|---|

## Slide 7
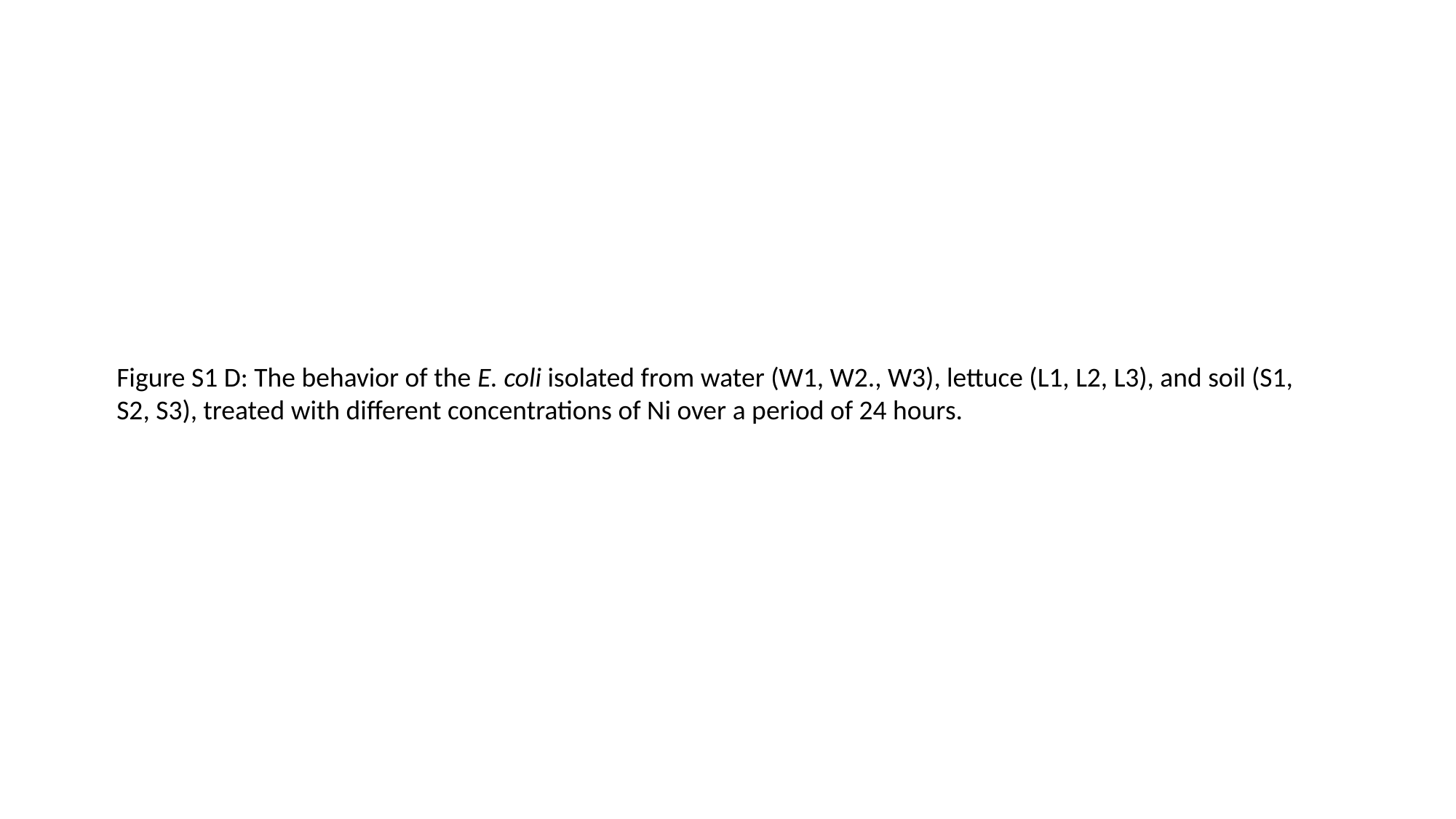

Figure S1 D: The behavior of the E. coli isolated from water (W1, W2., W3), lettuce (L1, L2, L3), and soil (S1, S2, S3), treated with different concentrations of Ni over a period of 24 hours.

## Slide 8
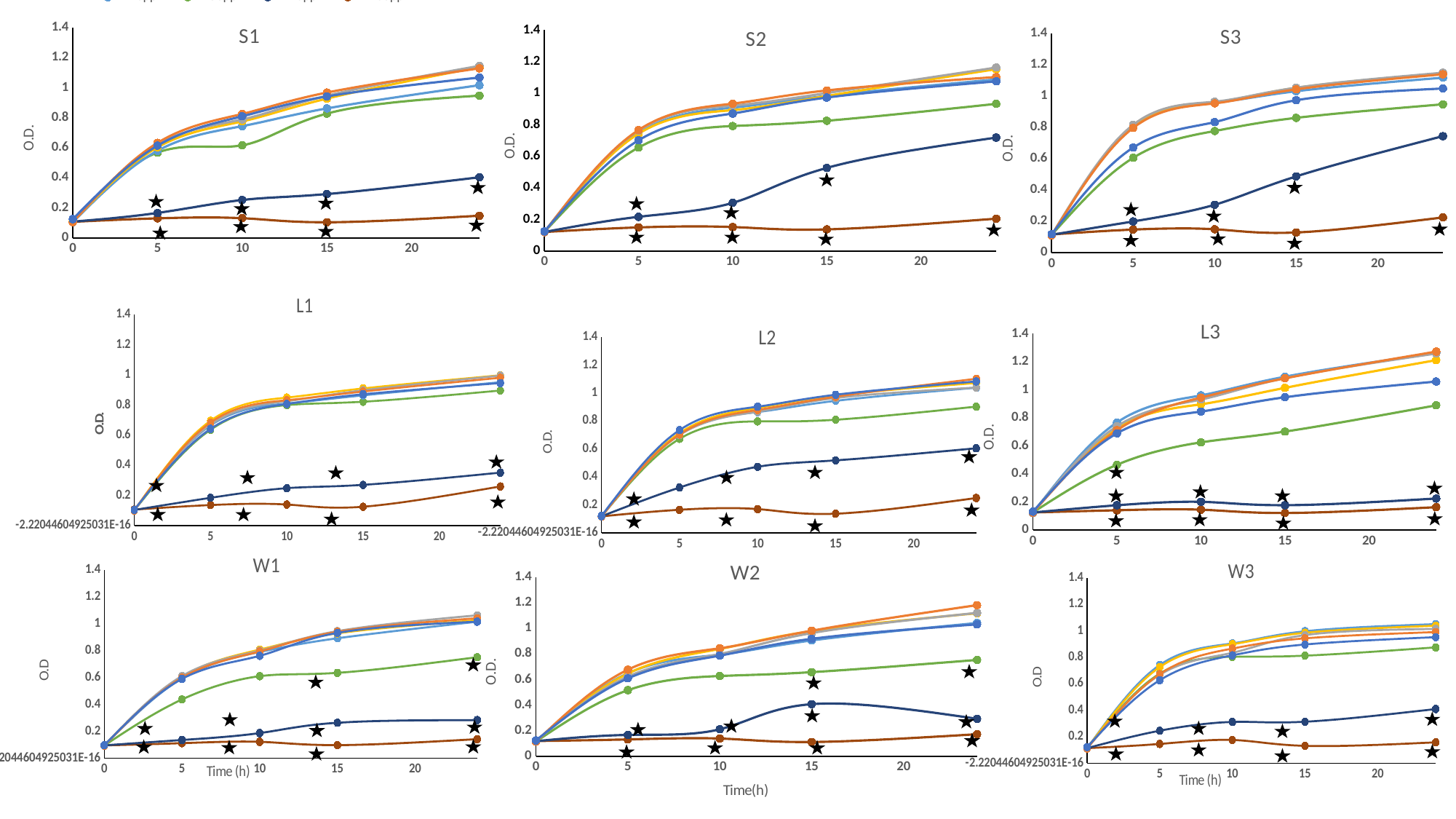

### Chart: S1
| Category | control | 1ppm | 5ppm | 10ppm | 20ppm | 50ppm | 70ppm | 100ppm |
|---|---|---|---|---|---|---|---|---|
### Chart: S3
| Category | control | 1ppm | 5ppm | 10ppm | 20ppm | 50ppm | 70ppm | 100ppm |
|---|---|---|---|---|---|---|---|---|
### Chart: S2
| Category | control | 1ppm | 5ppm | 10ppm | 20ppm | 50ppm | 70ppm | 100ppm |
|---|---|---|---|---|---|---|---|---|
### Chart: L1
| Category | control | 1ppm | 5ppm | 10ppm | 20ppm | 50ppm | 70ppm | 100ppm |
|---|---|---|---|---|---|---|---|---|
### Chart: L3
| Category | control | 1ppm | 5ppm | 10ppm | 20ppm | 50ppm | 70ppm | 100ppm |
|---|---|---|---|---|---|---|---|---|
### Chart: L2
| Category | control | 1ppm | 5ppm | 10ppm | 20ppm | 50ppm | 70ppm | 100ppm |
|---|---|---|---|---|---|---|---|---|
### Chart: W1
| Category | control | 1ppm | 5ppm | 10ppm | 20ppm | 50ppm | 70ppm | 100ppm |
|---|---|---|---|---|---|---|---|---|
### Chart: W3
| Category | control | 1ppm | 5ppm | 10ppm | 20ppm | 50ppm | 70ppm | 100ppm |
|---|---|---|---|---|---|---|---|---|
### Chart: W2
| Category | control | 1ppm | 5ppm | 10ppm | 20ppm | 50ppm | 70ppm | 100ppm |
|---|---|---|---|---|---|---|---|---|
