# Supplementary material for: Agricultural Mitigation Strategies to Reduce the Impact of Romaine Lettuce Contamination
Source: Plants (Basel). 2024 Sep 3;13(17):2460. doi: 10.3390/plants13172460 (PMC11396837; doi:10.3390/plants13172460)
Supplement: Supplementary file 1 [file plants-13-02460-s001.zip › Table S1.pdf]

Supplementary Table S1: Biological Absorption Coefficient (BAC) in lettuce leaves, pH, and Soil Organic Matter (SOM) o[illegible]soil in the five demo plots at the harvesting stage.

BAC	Ni	Zn	Cu	Mn	pH	SOM (%)
**D1**	1.18	8.39	11.52	4.86	7.4	2.75
**D2**	0.47	42.61	5.47	1.18	7.1	3.21
**D3**	1.13	6.79	4.45	0.93	7	2.18
**D4**	3.33	57.95	7.57	20.9	7.6	2.7
**D5**	6.41	14.09	4.35	21.25	7.6	1.7
